# FT-NIR Spectroscopy for the Non-Invasive Study of Binders and Multi-Layered Structures in Ancient Paintings: Artworks of the Lombard Renaissance as Case Studies

**DOI:** 10.3390/s22052052

**Published:** 2022-03-06

**Authors:** Margherita Longoni, Beatrice Genova, Alessia Marzanni, Daniela Melfi, Carlotta Beccaria, Silvia Bruni

**Affiliations:** 1 Dipartimento di Chimica, Università degli Studi di Milano, Via C. Golgi, 19, 20133 Milan, Italy; beatrice.genova@studenti.unimi.it (B.G.); alessia.marzanni@studenti.unimi.it (A.M.); daniela.melfi@studenti.unimi.it (D.M.); 2 Beccaria Carlotta & C. Studio Di Restauro, Via Conservatorio, 30, 20122 Milan, Italy; beccaria.restauro@gmail.com

**Keywords:** FT-NIR spectroscopy, binder, painting, stratigraphy, non-invasive analysis

## Abstract

This work deals with the identification of natural binders and the study of the complex stratigraphy in paintings using reflection FT-IR spectroscopy, a common diagnostic tool for cultural heritage materials thanks to its non-invasiveness. In particular, the potential of the near-infrared (NIR) spectral region, dominated by the absorption bands due to CH, CO, OH and NH functional groups, is successfully exploited to distinguish a lipid binder from a proteinaceous one, as well as the coexistence of the two media in laboratory-made model samples that simulate the complex multi-layered structure of a painting. The combination with multivariate analysis methods or with the calculation of indicative ratios between the intensity values of characteristic absorption bands is proposed to facilitate the interpretation of the spectral data. Furthermore, the greater penetration depth of NIR radiation is exploited to obtain information about the inner layers of the paintings, focusing in particular on the preparatory coatings of the supports. Finally, as proof of concept, FT-NIR analyses were also carried out on six paintings by artists working in Lombardy at the end of the 15th century, that exemplify different pictorial techniques.

## 1. Introduction

The analysis of the binders, i.e., the media in which the pigments are dispersed to be spread on the pictorial supports, is an important but demanding task in the field of conservation science.

Over the centuries, artists have used a wide range of materials—drying oils, natural gums and proteinaceous materials, such as egg white or yolk, animal glue or milk derivatives—associated with different painting techniques. Since ancient times, proteinaceous tempera based on egg yolk diluted in water was used in painting, while egg white or yolk, vegetable gums and animal glues were preferred, separately or in mixture, to create illuminated manuscripts. In the Renaissance, the use of drying oils spread in European painting and these gradually became one of the most used binding media during the modern age. The artists also experimented with recipes based on mixed binders to improve and modify their pictorial properties or to obtain particular chromatic effects by combining their different characteristics. This is the case, for example, of *tempera grassa*, an emulsion of oil and egg or, sometimes, oil and casein [1]. Furthermore, even the preparatory layers, i.e., the coatings spread on the support (typically a wooden panel or a canvas) before the application of the subsequent layers, represent an important aspect in the investigation of a painting. They were traditionally made of white mineral substances mixed with an adhesive, usually animal glue. Gypsum (CaSO_4_·2H_2_O) or calcite (CaCO_3_) were the main raw materials; the first was mostly adopted by artists from Mediterranean countries, while the second was typical of central-northern Europe [2,3].

In this scenario, the identification of the binders is of great interest both for the restoration and conservation of the paintings, and from the historical and artistic point of view. Until now, the analytical technique commonly used for their investigation is gas chromatography, often coupled with mass spectrometry (GC-MS) [4,5,6,7,8]. However, while being a very sensitive and specific method, it suffers from the main disadvantage of being destructive and sample-requiring. A completely non-invasive approach should instead be highly recommended in the field of cultural heritage. In any case, compared to inorganic pigments and fillers, the detection of binders is a difficult task as they are present in smaller amounts and more subject to chemical alterations. Referring instead to the study of the molecular composition of the preparatory layers and also of superimposed colour layers, it is feasible only when a sample containing the entire stratigraphy is available. Fourier-transform infrared micro-spectroscopy (micro-FTIR) in reflection mode can be used for this purpose, but it requires the preparation of a polished cross-section of the sample. GC-MS, on the other hand, does not usually allow for the discrimination of the layers, unless they are previously separated mechanically.

Among the spectroscopic techniques traditionally used for the non-invasive analysis of works of art [9,10,11,12,13,14,15], FTIR spectroscopy has acquired a key role thanks to the development of portable and compact instrumentation suitable for non-invasive in situ analyses in reflection mode. However, the mid-infrared (MIR) region (4000–400 cm^−1^), commonly studied to characterize materials, is dominated by the strong absorption bands of the pigments or, in general, of the substances that make up the surface layer of the painting, such as protective varnishes. On the other hand, the range from 7500 to 4000 cm^−1^ of the near-infrared (NIR) region shows interesting features for the identification of organic compounds. This spectral range is in fact dominated by overtone and combination bands, whose wavenumbers are multiples or sums of those of the fundamental bands—due to stretching or bending vibrations—which occur in the MIR. These absorption bands are broader and less resolved than the fundamental ones, but being orders of magnitude weaker, they allow the acquisition of reflection spectra that do not require any processing (e.g., Kramers–Kronig transform). Especially, the bands due to functional groups containing hydrogen atoms (e.g., OH, CH, NH) are found in the NIR part of the spectrum and can be exploited to identify organic binders, although their broadness can cause overlaps in the case of mixtures and superimpositions of different substances. This problem is generally addressed by using derivative methods, difference spectra and Fourier self-deconvolution, which allow to enhance the resolution [16,17]. Furthermore, the lower absorption coefficient and the higher energy of the NIR radiation allow a deeper penetration than MIR. This is a main advantage as, in principle, it allows to obtain information on the composition of the underlying layers.

For all these reasons, NIR spectroscopy has already been proposed for the identification of artistic binders [18,19,20,21], also pointing out that their spectral pattern in this region is not significantly affected by ageing and, therefore, fresh substances can be used as reference materials. In particular, Vagnini et al. [18] proposed first a spectral database of pure reference materials, highlighting the spectral features that can be useful to distinguish different compounds belonging to the classes of lipids, proteins or resins. However, these previous spectroscopic studies have mainly focused on pure binders. The NIR bands of organic binders were then exploited, for example, also in a reflectance imaging investigation of illuminated manuscripts to identify the presence of a generic binder containing fatty acids [22]. The present work instead intended to investigate the possibility of exploiting FT-NIR spectroscopy to recognize specific binders, such as drying oils, egg yolk or even their mixtures, in complex multi-layered systems, as often were ancient easel paintings. In this regard, the potential usefulness of the deeper penetration of NIR radiation to obtain information on the inner layers of a painting was also considered. To this end, mock-up painting samples were first made in the laboratory following ancient recipes and using different binders to spread pigments in one or more layers on wooden panels. In particular, inorganic pigments showing absorption bands in the NIR region were chosen in order to evaluate how they can influence the identification of binders. Two different ground layers were used for the models, respectively consisting of calcite or gypsum mixed with animal glue, considering that, unlike calcite, gypsum has characteristic absorptions in this spectral region. Finally, a multivariate approach based on principal component analysis (PCA) of spectral data was also investigated as a possible tool to differentiate binders.

As a proof of concept, FT-NIR analyses were then carried out on ancient paintings, to gain a deeper insight on the applicability of this method for the non-invasive detection of binders and the stratigraphic study of paintings. Works of art belonging to the Lombard Renaissance were examined, in particular a “Madonna and Child” by Giovanni Antonio Boltraffio (ca. 1495), a “Nursing Madonna” by an anonymous Lombard school painter (ca. 1500–1510), again a “Madonna with Child” by Francesco Galli, also known as Francesco Napoletano (ca. 1490–1500), “Ecce Homo” by Andrea Solario (1503–1505), “St. John the Baptist” by Bernardo Zenale (ca. 1490–1520) and “St. Augustine and St. Jerome” by Ambrogio da Fossano known as Bergognone (1492–1494). These paintings are particularly interesting from the point of view of the investigation of binders, as they belong to the transition period from the use of tempera to that of drying oil. Another interesting feature is that, even if Italian paintings of the time had in many cases a preparatory layer made of gypsum, in other cases, it was not present or was replaced by an oil priming layer which could contain different pigments [23].

## 2. Materials and Methods

### 2.1. Materials

Linseed and walnut oil were purchased from local paint shops in Milano (Italy). Lead white, azurite, yellow ochre, lead tin yellow and dammar resin were purchased from Zecchi (Firenze, Italy). Red lead and hematite were bought from Sigma Aldrich.

### 2.2. Instrumentation

A Bruker Alpha FTIR spectrophotometer was used for non-invasive analyses in reflection mode. The instrument is equipped with a reflection module for contactless measurements and a deuterated triglycine sulfate (DTGS) detector, operating at room temperature and ensuring a linear response in the spectral range between 7500 and 375 cm^−1^. The FTIR spectrophotometer collects spectra from a sample area with a diameter of approximately 6 mm and with a resolution of 4 cm^−1^. An integrated camera allows the operator to select the area to be measured. FTIR spectra were acquired as sum of 200 scans after the background spectrum acquisition on a gold mirror. The reflection spectra in the MIR region were processed by the Kramers–Kronig transform using the Bruker OPUS software. In the NIR range, on the other hand, they were transformed into pseudo-absorbance Log(1/R).

### 2.3. Sample Preparation

#### 2.3.1. Pure Binders

The pure reference binders were spread directly on glass slides and left to dry in the air or, especially in the case of drying oils, in an oven set at ≈100 °C for 72 h to accelerate the polymerization process. In particular, the reference materials were siccative oils (linseed, poppy and walnut), egg (white and yolk) and animal glue.

#### 2.3.2. Mock-Up Painting Samples

**Figure 1:**
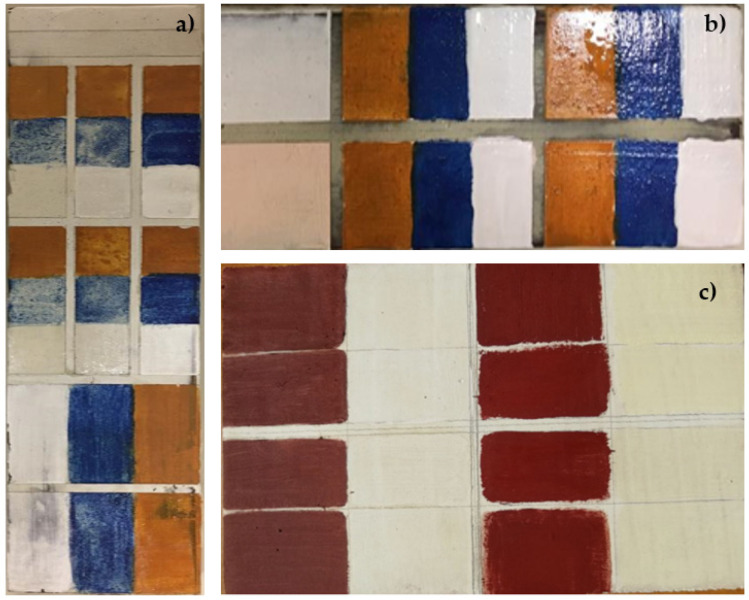
Mock-up samples prepared in the laboratory: painting layers on (**a**) calcite ground layer; (**b**) white and pink priming layers; (**c**) gypsum ground layer. The pigments were lead white, azurite and yellow ochre for (**a**) and (**b**), and lead white and hematite for (**c**).

Mock-up samples, simulating the complex layered structure of real paintings (Figure 1), were prepared on the basis of ancient treatises [24] and especially of the literature reporting the results of scientific studies on Old Masters’ paintings dating to the 15th–16th century [23,25,26]. They were made on a wooden panel, and two different preparatory layers were used, commonly adopted by artists, and respectively based on calcite and gypsum.

In the first case, a ground layer made of calcium carbonate (CaCO_3_) mixed with rabbit skin glue (ratio 1:7) was first applied to the wooden support. The mock-up samples were then prepared according to the stratigraphy reported in Table 1. The selected inorganic pigments were yellow ochre FeO(OH)·nH_2_O, azurite 2CuCO_3_·Cu(OH)_2_ and lead white 2PbCO_3_·Pb(OH)_2_, which all show absorption bands in the NIR region that may overlap with those of the binders. The binding media chosen were linseed or walnut oil, egg yolk and a mixture of the two to produce the so-called *tempera grassa*. Two identical series of samples were prepared, and a final layer of dammar varnish was applied to one of them. Egg tempera was prepared from egg yolk, previously rinsed with water and then pierced with a wooden stick to allow the spilling of the liquid used as binder. A spoonful of vinegar was added to avoid fermentation. Both the drying oils and the egg yolk were mixed with the pigments in a ratio of approximately 1:1. Finally, the varnish was obtained by dissolving the dammar resin in turpentine essence.

For the second series of models, a preparatory layer of gypsum (CaSO_4_ 2H_2_O) mixed with rabbit glue (ratio 2:1) was first applied to the wooden panel, followed by a second (ratio 1:1) and a third one with a higher proportion of glue and diluted with water (ratio 1:2). Two different pigments (lead white and red ochre Fe_2_O_3_) with oil or tempera binders, pure, in mixture or in multiple layers, were then applied on this coating (see Table 1).

### 2.4. Preparation of Cross-Sections

Cross-sections were prepared from the mock-up samples to evaluate the thickness of the paint layers and correlate it to the penetration depth of the NIR radiation. For each sample, a small fragment containing all layers was carefully collected by a scalpel and placed in a silicon mold on an already hardened layer of the embedding resin. The latter was prepared by mixing Struers Epoxy resin and Epofix hardener in a 15:2 ratio and stirring until the mixture was completely homogenous. The sample was then covered with a second layer of resin and left to cure overnight. The cross-sections were manually polished using a Struers Labopol-1 lapping machine equipped with a magnetic rotating disk. Several silicon carbide abrasive papers with decreasing granulometry were used in succession, until the surface of the sample came out of the resin block. A 6-µm DP-Struers diamond paste was then used to obtain a perfectly smooth surface. Finally, the cross-sections were observed and photographed using an Olympus optical microscope.

### 2.5. Data Processing

The FT-NIR spectra of the mock-up samples and pure binders were processed by principal component analysis (PCA), performed by the MINITAB 14 software. The spectra were first truncated between 6000 and 4000 cm^−1^ and normalized between 0 and 1. Subsequently, they were transformed into the corresponding 1st derivatives using GRAMS AI software (Thermo Fisher Scientific) and the Savitsky–Golay algorithm (2nd degree polynomial and 31 points), to eliminate the contribution of the baseline slope and to emphasize the differences among the spectra. The variables to which PCA analysis is applied are, therefore, the dA/d$\bar{\nu }$ values in the range 6000–4000 cm^−1^. The covariance matrix was used to give less weight to baseline points in the calculation. 

To calculate the ratios between band intensities, the baseline was subtracted from the spectrum and the band heights were evaluated by using the Grams/AI software. Absorption bands typical of oil or egg yolk, respectively, were chosen to exploit the ratio as discriminating value between the two binders (see Section 3.3 for more details). When bands due to the gypsum ground layer were observed in the NIR spectrum besides those of the binder, the contribution of calcium sulphate dihydrate was subtracted using the same software.

### 2.6. Case Studies

As proof of the concept of the proposed methodology, six paintings dating back to the Lombard Renaissance were studied using FT-NIR spectroscopy. Three of the paintings under investigation were made in the last two decades of the 15th century by pupils of Leonardo da Vinci. They were the “Madonna and Child (Madonna of the Rose)” by Giovanni Antonio Boltraffio and the “Madonna nursing the Child” by an unknown Lombard school painter, exhibited at the Poldi Pezzoli Museum in Milan, and the “Madonna and Child” by Francesco Galli, called Francesco Napoletano, stored at the Pinacoteca di Brera in Milan. These masterpieces have already been extensively characterized using imaging and non-invasive spectroscopic techniques, as part of a dedicated project coordinated by the Italian Consiglio Nazionale delle Ricerche—CNR and the University of Milano-Bicocca [27], and the results of FT-NIR measurements reported here were collected during that survey campaign. Two other paintings were examined on the occasion of their restoration, namely “St. Augustine and St. Jerome”, painted at the end of the 15th century by Ambrogio da Fossano, known as Bergognone, and belonging to the Chartreuse of Pavia, and “St. John the Baptist”, a work by Bernardo Zenale from the beginning of the 16th century and part of a private collection. All the above works are painted on a wooden panel. Finally, during its restoration, the coeval painting “Ecce homo”, made by Andrea Solario on a paper support applied on a panel and belonging to the Accademia Carrara in Bergamo, was also analysed for comparison.

## 3. Results and Discussion

### 3.1. Model Samples with CaCO_3_ Preparatory Layer

Since calcium carbonate does not absorb in the NIR region, the only signals observed in this range for the ground layer spectrum for these model samples are due to the animal glue used to prepare it (Supplementary Materials Figure S1). The NIR spectra obtained for the different painting layers will be discussed in detail below.

**Figure 2:**
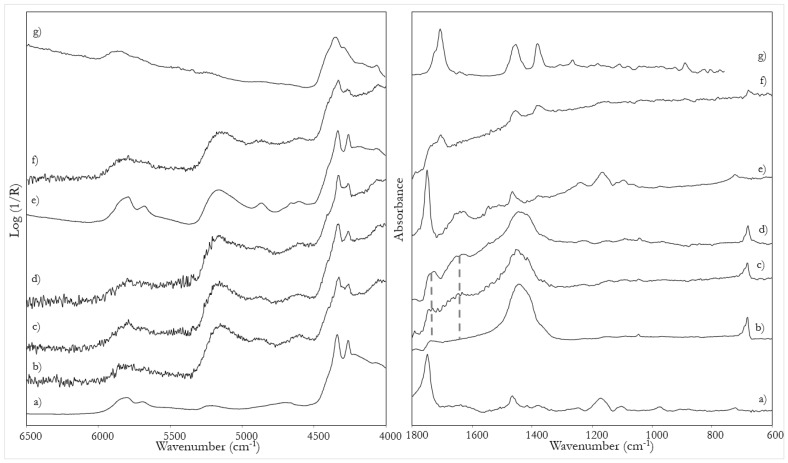
NIR (left) and MIR (right) spectra of: (**a**) linseed oil; (**b**) model painting sample of linseed oil on tempera; (**c**) model painting sample of *tempera grassa*; (**d**) model painting sample of tempera; (**e**) egg yolk; (**f**) model painting sample of *tempera grassa* with dammar varnish; (**g**) dammar resin. All model samples contained lead white as a pigment. All spectra of the reference materials and model painting samples were acquired in reflection mode, with the exception of the spectrum of dammar which was acquired in transmission mode. The dashed lines indicate the two bands at 1740 and 1650 cm^−1^, respectively characteristic of oil and yolk in the MIR region.

*Tempera*: The NIR spectra of all tempera mock-up samples (Figure 2d) allow the identification of the proteinaceous binder, thanks to the characteristic bands at 5793, 5680, 5160, 4860, 4604, 4330 and 4258 cm^−1^, clearly recognizable in the spectrum of pure yolk (Figure 2e). The corresponding assignments are reported in Table 2. All inorganic pigments used show signals in this spectral range, because of the presence of -OH groups in their chemical structure. In particular, lead white is recognizable from the band at 4275 cm^−1^, azurite from those at 4370 and 4245 cm^−1^ and yellow ochre at 4526 cm^−1^ [14,28,29] (see Figure 2 for the spectra of pictorial models containing lead white and Figures S2 and S3 and Table S1, for those containing the other pigments).

Furthermore, some minor spectral features suggest a contribution of the glue from the preparatory and the priming layers, in particular, the slight broadening of the band at 4860 cm^−1^ and the shoulder at about 4400 cm^−1^ (Table 2). The MIR region of the spectra is dominated by the signals of the inorganic pigments (for lead white at 1443 and 683 cm^−1^), while the binder is suggested only by the bands at about 1650 and 1740 cm^−1^, respectively, due to the proteinaceous and lipidic components of yolk (Table 2).

*Tempera grassa:* When the binder is a mixture of yolk and a smaller amount of linseed oil, the NIR spectra (Figure 2c) are dominated by signals due to the first substance. In any case, there is an increase in the signals at about 4330 and 4258 cm^−1^ (Figure S4), typical of the lipidic component (Table 2). Furthermore, also in the MIR region the relative intensity of the broad band at 1650 cm^−1^ compared to the signal at 1740 cm^−1^ is slightly higher in the case of the pure egg binder than for the mixture with oil (Figure 2c).

*Oil on tempera*: The spectra of the superimposed layers of oil on tempera are very similar to those of *tempera grassa* in the NIR region. However, focusing again on the MIR, the band at 1650 cm^−1^ disappears while the one at 1740 cm^−1^, typical of siccative oils, is more evident (Figure 2b). Therefore, by combining the information from the NIR and MIR spectra, it is possible to distinguish a mixture from an overlap of oil and tempera binders. This is a proof of the different penetration depths of NIR and MIR radiation, which can therefore be exploited for a stratigraphic investigation.

*Dammar varnish on tempera grassa*: When a layer of dammar resin is applied as varnish on the paint samples, the MIR spectra are dominated by its signals (at 1705, 1455 and 1380 cm^−1^), which hide those of the inorganic pigments, as shown in Figure 2f for a sample of *tempera grassa*. In the NIR range, on the other hand, the spectral trend remains substantially unchanged (except for a slight broadening of the signal at 4330 cm^−1^), allowing the identification of the binder. This fact is, once again, explained by the greater penetration depth of the NIR radiation, which can pass through the most superficial layer. The cross-section obtained from a small fragment of the varnished mock-up sample allowed one to estimate a thickness of 80 μm for the surface coating (Figure S5).

**Figure 3:**
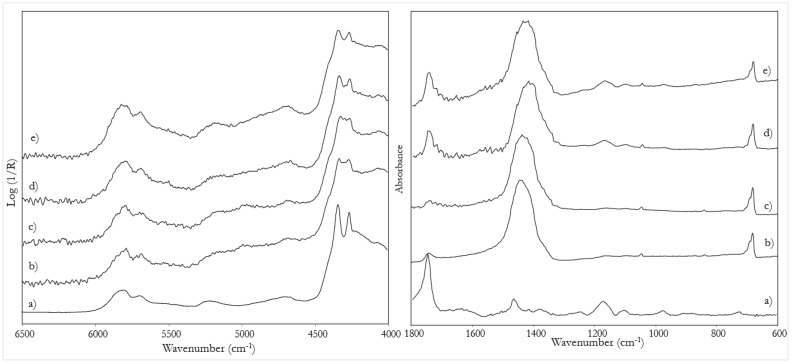
NIR (**left**) and MIR (**right**) spectra of: (**a**) linseed oil, (**b**) white priming layer, (**c**) pink priming layer, (**d**) lead white in linseed oil on white priming layer, (**e**) lead white in linseed oil on pink priming layer. All spectra of reference materials and model painting samples were acquired in reflection mode.

*Oil on oil priming layer*: The NIR spectra acquired from these mock-up samples clearly demonstrate the presence of drying oil as a binder (Figure 3). The bands at 5805, 5690, 5208, 4694, 4340 and 4260 cm^−1^ are, in fact, typical of lipidic substances (Table 2). The spectra of the two priming layers are very similar to each other (Figure 3b,c), confirming that the presence of the two red lead and lead tin yellow inorganic pigments in the pink sample do not affect the spectrum in the NIR region. In both cases, the signal already mentioned at 4275 cm^−1^ and a weaker one at 5135 cm^−1^ are due to lead white. Such signals are less evident when a painting layer, even containing the same pigment, is applied on the primer, as the latter contains a higher pigment to binder ratio than the former (Figure 3d,e). The MIR region is instead dominated by the signals due to the inorganic pigment, associated with the characteristic drying oil band at 1740 cm^−1^ (Figure 3). The spectra of pictorial models containing yellow ochre and azurite are shown in Figures S6 and S7, and the corresponding assignments are reported in Table S1.

**Figure 4:**
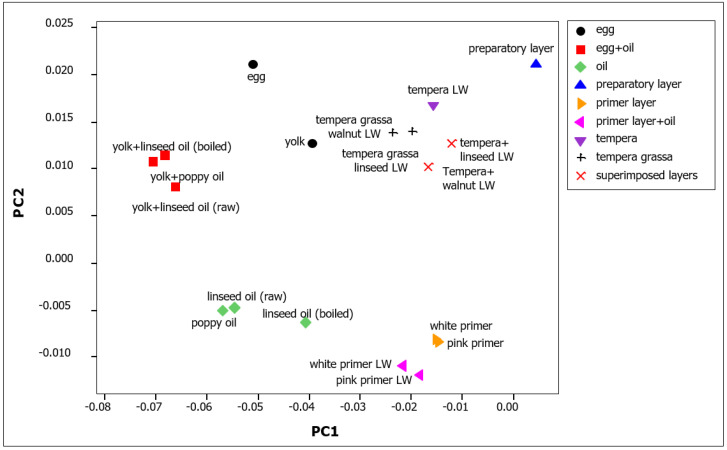
Score plot of the first two principal components of the NIR spectra (6000–4000 cm^−1^) of pure binders and of model painting samples on calcite ground layer. In the model samples, lead white (LW) was used as pigment.

Principal component analysis (PCA) applied to NIR spectra in the 6000–4000 cm^−1^ region of pure binding media (alone or mixed) and of the painting mock-ups confirms the differences and similarities among the spectra already discussed above. To give greater consistency to the statistical analysis, in addition to linseed oil, poppy and walnut oils were also included in the PCA, both as pure binders and in pictorial models. As shown in the upper part of the score plot (Figure 4) for the models with lead white, when there is coexistence of oil and tempera, the spectra match the group of the mixed binders. However, there is a shift towards the symbol corresponding to the ground layer because of the slight variation of the bands caused by the presence of rabbit glue. Indeed, in the mock-ups, the painting layer is thin enough (40–60 µm) to allow NIR radiation to penetrate and reach the preparatory layer. On the other hand, both the priming layers (white and pink), with or without a superimposed layer of lead white in oil, occupy the region corresponding to the group of pure oils in the graph. The loadings of the first two principal components for these spectra are reported as supplementary material (Figure S8). The loadings of the first principal component correspond mainly to the opposite of the first derivative of the spectrum of a siccative oil, possibly containing also a contribution from the spectrum of lead white. Instead, the loadings of the second principal component are dominated by features corresponding to those of egg yolk. In conclusion, PCA is a powerful method for highlighting the differences between these spectra. For this reason, even if similar results were obtained for all the three investigated pigments (Figures S9 and S10), the corresponding painting layers must be considered separately because of the presence of their characteristic signals in the NIR range.

### 3.2. Model Samples with CaSO_4_·2H_2_O Preparatory Layer

A further step was the study of the influence in the NIR spectra of a preparatory layer made of gypsum. Indeed, this material is characterized by an intense absorption in this region, due to the two water molecules in its chemical structure. In particular, the spectrum of calcium sulphate dihydrate shows a strong band at 5150 cm^−1^ with a shoulder at 5060 cm^−1^, and minor combination bands at 4515 and 4405 cm^−1^ (Table 2). In the present study, the possibility of identifying the binder through the NIR spectrum was investigated even in the presence of gypsum and the information was correlated with the thickness of the painting layer as estimated from the corresponding cross sections. For this purpose, models with one and two layers of colour on the gypsum ground layer were prepared.

**Figure 5:**
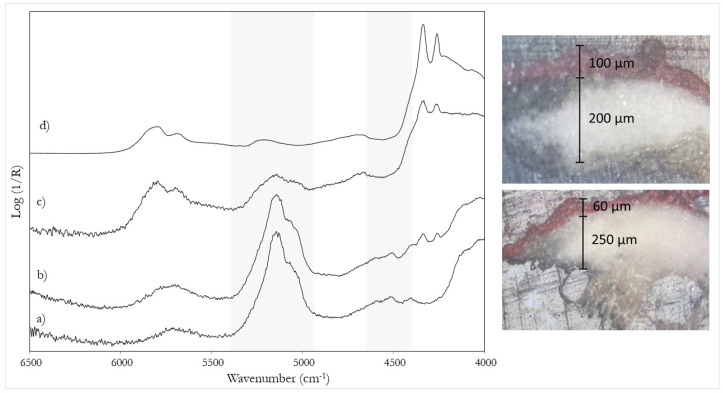
NIR spectra of: (**a**) gypsum preparatory layer; (**b**) a layer of oil paint on gypsum; (**c**) two layers of oil paint on gypsum; (**d**) linseed oil. The bands due to gypsum are highlighted. Cross-sections of mock-up samples corresponding respectively to (**b**) (bottom) and (**c**) (top) are also shown.

In general, when a single colour layer is applied, with a thickness around 60 μm, the NIR spectrum is dominated by gypsum signals, preventing the binder from being identified (Figure 5b). When a second layer of colour is applied (for a total thickness of about 100 μm), the contribution of calcium sulphate dihydrate decreases and that of the binder becomes more evident (Figure 5c). In this situation, the discrimination of a tempera from an oil and even of a mixed binder becomes possible thanks to the signals due to the combination bands at 4860 and 4604 cm^−1^, typical of the proteinaceous component.

**Figure 6:**
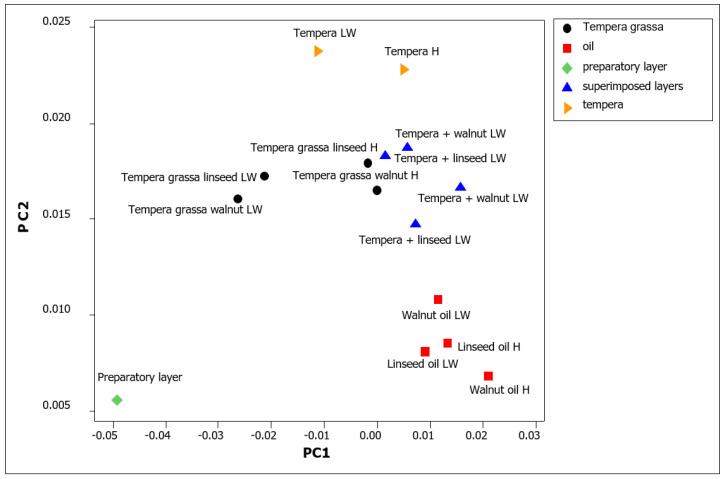
Score plot of the first two principal components of the NIR spectra (6000–4000 cm^−1^) of model painting samples on gypsum ground layer. The binders are tempera or linseed oil, used pure, in mixture or in superimposed layers. The pigments are lead white (LW) and hematite (H).

This fact is again evidenced by the PCA score plot of the NIR spectra in the range 6000–4000 cm^−1^ of these model samples (Figure 6). The loadings of the first two principal components of these spectra are also reported as supplementary material (Figure S11). The loadings of the first principal component correspond mainly to the first derivative of the spectrum of gypsum itself, while the loadings of the second component show again the typical pattern of egg yolk. It should be emphasized that, in all the model samples considered in the score plot, the thickness of the colour layer was similar, so that the contribution of the gypsum bands to the spectra was also comparable. To appreciate the effect of this contribution, it can be observed that in the plot the points associated with layers with a mixed binder are displaced along the first component towards the point of the spectrum of the ground layer, dominated by the bands due to gypsum. In those models, in fact, the thickness of the colour layer is slightly less than in the painting mock-ups in which two layers with different binders were superimposed.

In summary, the greater penetration depth of NIR radiation allows the identification of gypsum in the preparatory layer, as already known [34]. The bands due to gypsum, on the other hand, can have varying intensities in the spectra depending on the thickness of the overlapping colour layers. In principle, this fact could hinder the application of PCA analysis of spectral data in the 6000–4000 cm^−1^ region to the identification of binders and of their mixtures.

### 3.3. Case Studies

**Figure 7:**
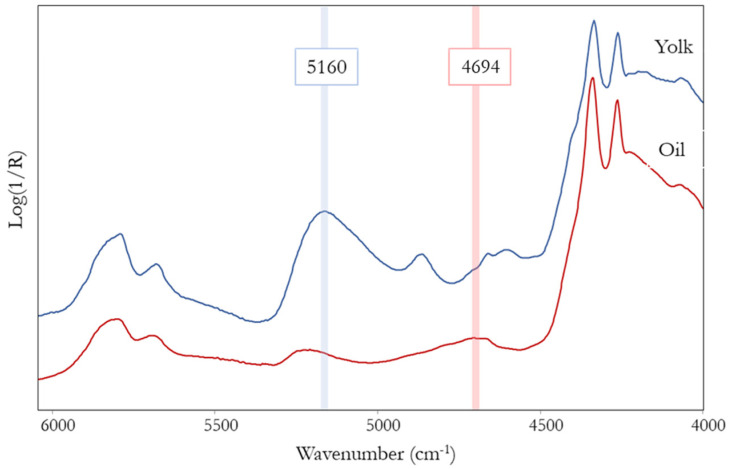
Highlighting of the absorption bands at 4694 and 5160 cm^−1^ typical of oil and egg yolk, respectively, and chosen for the calculation of the intensity ratio used to distinguish the two binders and their possible mixture.

Based on the encouraging results obtained on model painting samples, FT-NIR spectroscopy was then applied to the investigation of binders and preparatory layers in the ancient masterpieces listed above. It is worth noting that in a real painting, the situation is more challenging than in mock-up samples: the impossibility of predicting the stratigraphy, both in terms of composition and thickness of the layers, and the eventual presence of degradation products make the interpretation of NIR spectra more difficult. Calcium oxalates and metal soaps, for example, are typical degradation products formed by the interaction between a drying oil and metal ions, e.g., Ca^2+^, Pb^2+^ or Zn^2+^, contained in the pigments. These compounds are characterized by absorption bands in the NIR range that can interfere with those of the binder. In particular, whewellite and weddellite (the mono and dihydrate forms of calcium oxalate, respectively) have intense bands in the range 6650–6250 and 5550–4500 cm^−1^ [34]. The NIR spectrum of a metal soap (i.e., lead palmitate) is instead close to that of an oil: their chemical structure is, in fact, very similar as they are compounds containing heavy metals combined with carboxylic acids of 7 to 22 carbon atoms. The spectrum of lead palmitate was acquired as a reference (Figure S12) after having synthesized the compound as described in [35]. Due to these uncontrollable factors, the use of PCA for the identification of binders in the ancient paintings failed. In fact, being very sensitive, this method of data analysis is also strongly affected by subtle differences among the spectra, as already noted above. For these reasons, an alternative method is proposed which can be combined with the observation of the spectral pattern. It is based on the calculation of the ratio between the absorbance values at 4694 and 5160 cm^−1^, where typical bands are found, respectively, for oil and tempera (Figure 7).

**Figure 8:**
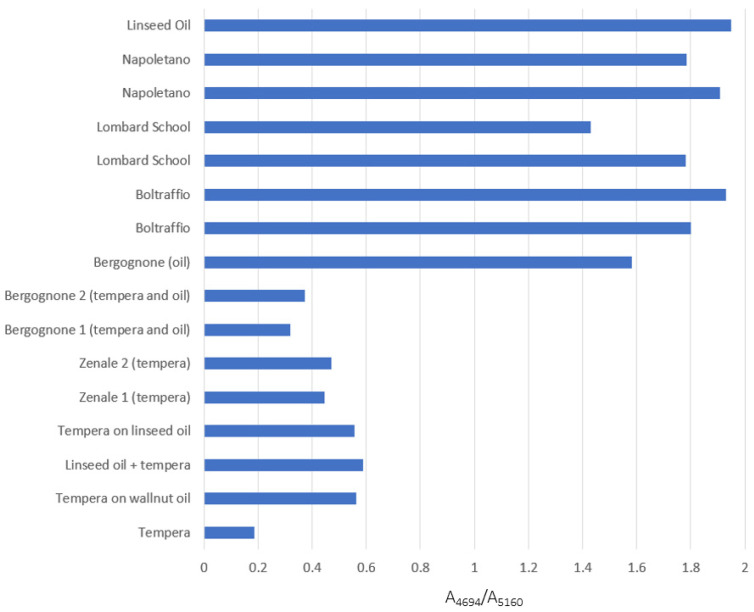
Histogram summarizing the values of the ratio between the absorbance values at 4694 and 5160 cm^−1^, calculated for the reference samples and for the ancient paintings examined.

**Figure 9:**
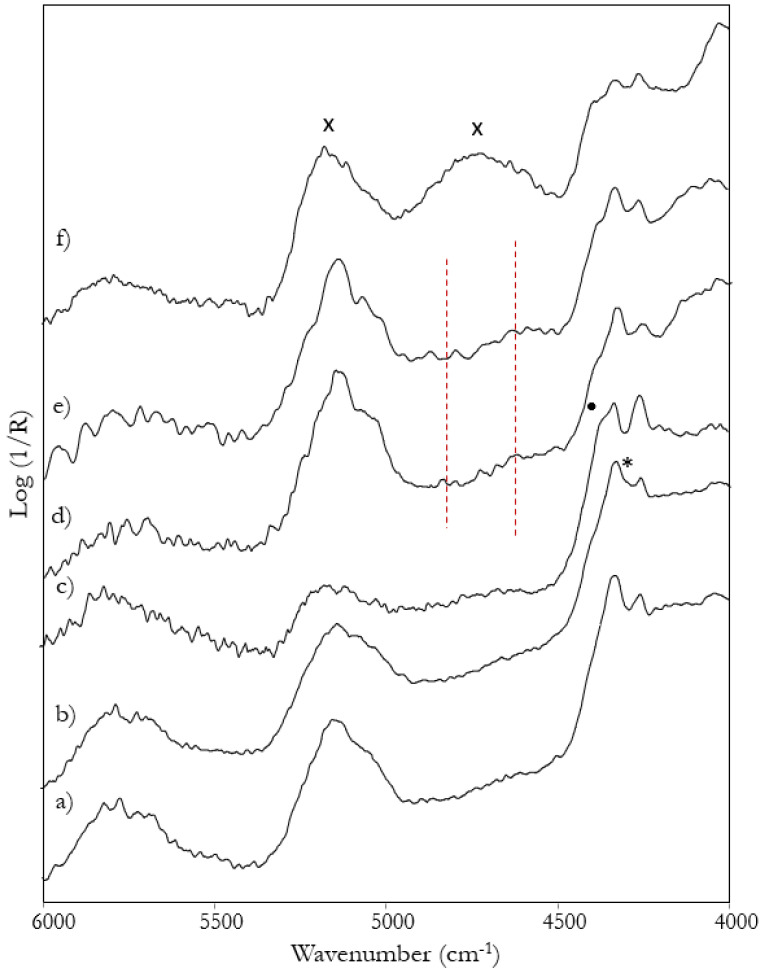
FT-NIR spectra from some details of the paintings (**a**) “Madonna and Child” by G. A. Boltraffio; (**b**) “Madonna and Child” by F. Galli; (**c**) “Madonna nursing the Child” by Lombard school painter; (**d**) “St. Augustine and St. Jerome” by Bergognone; (**e**) “St. John the Baptist” by B. Zenale. The red dashed lines mark the weak bands associated with egg tempera. Bands due to paper in spectrum (**f**) are marked with “x”.

The ratio of their values was found to be in the range 0.2–0.5 for pure tempera, between 0.5 and 0.8 when the two binders are mixed or in overlapping layers, and greater than 1 for pure oil. The effectiveness of the method was successfully verified for the mock-up samples before applying it to the cases of study. The results obtained both for models and for ancient paintings are summarized in the histogram of Figure 8, and are confirmed by the observation of the spectral pattern (Figure 9) and by the literature data available for the paintings. It is worth noting that all the ancient works analysed had a thin finishing layer of varnish applied after their restoration, with the exception of “Ecce Homo” by Solario. As demonstrated also experimentally by comparing the NIR spectrum of an oil film as such and with a layer of synthetic varnish superimposed (Figure S13), it is, however, possible with this technique to gain information on the original binders of the painting, again thanks to the penetration depth of NIR radiation. As expected, instead, in the FT-MIR spectra of the same areas of the paintings (Figure S14), only the bands due to the varnish could be observed.

In detail, in the “Madonna and Child” by Giovanni Antonio Boltraffio [36] FT-NIR analyses (Figure 9a) revealed the presence of a gypsum preparatory layer (as already reported in [14]), while the binder is oil as suggested by the values (1.8 and 1.9) of the ratio calculated as described in Section 2.5 for two details of the painting, namely the Child’s hair and the Virgin’s complexion. Similar results were obtained for all the areas analysed in this painting. These results agree with those of a previous analysis campaign conducted in the National Gallery laboratories by means of microdestructive techniques. GC-MS analyses have in fact identified oil as binder, while micro-analyses on a cross-section of the painting have shown the presence of a gypsum ground, covered by a 60-µm thick colour layer [37]. The possibility of detecting the gypsum preparatory layer by the NIR analysis is therefore consistent with what observed on mock-up samples. Similar results were obtained for the “Madonna and Child” by Francesco Galli [38] (Figure 9b), for which gypsum was identified in the ground layer (see also [27]) and it was possible to demonstrate the use of a drying oil as a binder, consistent with the absorbance ratio values (1.4 and 1.7) calculated for two measurement areas (the Virgin’s shoulder and her red dress) as an example.

As for the “Madonna nursing the Child” by an anonymous painter of the Lombard school [39], the NIR spectra (Figure 9c) indicate that gypsum was not used in the ground layer (see also [14]), in accordance with previous microdestructive analyses [40], while the ratio calculated for two areas of the painting corresponding to the Virgin’s blue dress and to the light blue sky (values of 1.7 and 1.9, respectively) still suggests that a siccative oil was used as a binder.

A different situation occurred for some details of the paintings by Bergognone [41] (the white fur and the blue of the border) and Zenale [42] (the banner held by the Saint and the sky), both characterized by a preparatory layer containing gypsum (Figure 9d,e). In fact, in other areas of the two artworks just oil was recognised as a binder, but for the above-mentioned details, the absorbance ratio values between 0.3 and 0.4 suggest the coexistence of tempera and oil in those areas of the paintings. It was thus possible to support by the NIR spectroscopic analyses the hypothesis that had been advanced during the restoration works. In fact, the visual inspection of the paintings showed that some areas had a more compact and covering texture, and in these areas both oil and tempera were detected by NIR analyses. On the contrary, areas where only oil was recognised as a binder appeared more transparent. The fact that the two paintings had a synthetic varnish as a surface coating applied during the restoration prevented the comparison with the MIR spectra and, consequently, the possible determination of whether the two binders were mixed or contained in overlapping layers (as described in Section 3.1). It should be emphasised that usually the original varnish is removed during the restoration and, therefore, if the paintings could be analysed after this step and before the application of the modern final coating, the distinction should again be possible, at least in principle.

Finally, a separate case is represented by “Ecce Homo” painted on paper by Andrea Solario [43]. In the NIR region the spectra (Figure 9f) are dominated by the bands at 5180, 4745, 4390 and 4280 cm^−1^, typical of cellulose [44] and, therefore, of the paper that constitutes the support of the painting. For some details, it is also possible to distinguish the two most intense peaks at about 4330 and 4265 cm^−1^ of a lipidic component, but the prevalence of the signals associated with the support prevents the observation of bands possibly due to the proteinaceous part of a tempera binder. Therefore, based on the measurements made and thanks to the penetration depth of the NIR radiation, it can be assumed that both a gypsum preparatory ground and a possible oil priming are absent in this painting, while a definite answer cannot be given as regards the binder, unlike the above-discussed coeval paintings on wooden panel.

## 4. Conclusions

In the present work, the potential of FT-NIR spectroscopy for the non-invasive characterization of binders and preparatory layers directly on the paintings was investigated.

Complex model samples were prepared, with two different compositions of the preparatory layer, gypsum or calcite, and the use of drying oil and egg tempera binders, separately, in mixture (*tempera grassa*) and in overlapping layers. Various pigments were also used in the models, including pigments containing hydroxyl groups and therefore prone to absorb NIR radiation. A multivariate approach based on principal component analysis (PCA) was used to emphasize the subtle differences in the spectral patterns, allowing the identification not only of oil and tempera, but also of *tempera grassa* and of superimposed layers containing the two binders. The possibility of identifying the presence of gypsum in the ground layer was related to the thickness of the overlying colour layer.

Finally, FT-NIR analyses were carried out on six paintings by artists operating in Lombardy at the end of the 15th century. PCA analysis did not allow a fully satisfying comparison between ancient paintings and model samples, as the even small differences between their spectra (due to ageing and, possibly, alteration products) cause a separation in the score plot. Therefore, a different approach was suggested, based on the observation of the spectral pattern and the calculation of the intensity ratio between characteristic bands of oil and egg tempera. It was thus possible to provide an insight into the applicability of this method for the non-invasive detection of binders, even if different ones were used in the same painting. At the same time, information could be obtained on the presence or absence of a preparatory layer, in particular a gypsum-based one, or of a priming layer.

In fact, when such layer is composed of gypsum, it is easily recognised, as can be seen in the paintings of Boltraffio, Napoletano, Bergognone and Zenale; in the absence of both the ground layer and a primer, characteristic bands of the support are observed, as seen for the Solario painting on paper, unlike the case of the painting by the anonymous artist of the Lombard school, where the gypsum ground layer is absent but the presence of an oil priming layer hides the bands due to the support.

Even when the non-invasive approach proposed in the present work cannot provide as much specific information on binders as destructive chromatographic analyses, it still allows for a screening of the pictorial surface which can help in the choice of representative areas for possible sampling.

## Figures and Tables

**Table 1:** Stratigraphy of the mock-up samples realized on a wooden panel.

Preparatory Layer	Priming Layer ^a^	Binders	Pigments
CaCO_3_ + animal glue	Animal glue	Egg tempera	Yellow ochre Lead white Azurite
		*Tempera grassa*	
		Linseed oil on tempera	
	Walnut oil + lead white	Linseed oil	
	Walnut oil + lead white + red lead + lead tin yellow		
CaSO_4_·2H_2_O + animal glue	-	Linseed oil	Hematite Lead white
		Walnut oil	
		Egg tempera	
		*Tempera grassa*	
		Linseed oil on tempera	
		Tempera on linseed oil	

^a^ A primer layer was not applied over the gypsum ground, but more coats were applied (see text) for each pigment spread in oil.

**Table 2:** Assignments of the main bands observed in the NIR and MIR spectra of the components of the mock-up samples.

NIR (6000–4000 cm^−1^)			MIR (1800–400 cm^−1^)	
Drying oil [18,30,31]	4260	ν_s_(CH_2_) + δ(CH_2_)	1746	ν(C = O) triglyceride ester
	4340	ν_a_(CH_2_) + δ(CH_2_)	1466, 1380	δ(C–H)
	4694	ν(C = O) + ν_a_(CH_2_)	1246, 1172, 1102	ν(C–O)
	5208	2nd overtone ν(C = O) ester	724	C−H torsion band
	5690	1st overtone ν_s_(CH_2_)		
	5805	1st overtone ν_a_(CH_2_)		
Yolk [18,30,32]	4258	ν_s_(CH_2_) + δ(CH_2_)	1746	ν(C = O) triglyceride ester
	4331	ν_a_(CH_2_) + δ(CH_2_)	1654	ν(C = O) amide I
	4602	ν(NH) + amide II	1542	δ(C–N–H) amide II
	4660	-	1460, 1380	δ(C–H)
	4865	ν(NH) + δ(NH)	1238, 1164, 1065	ν(C–O) triglyceride ester
	5165	ν(OH) + δ(OH)		
	5680	1st overtone ν_s_(CH_2_)		
	5793	1st overtone ν_a_(CH_2_)		
Animal glue [18,32]	4257	ν_s_(CH_2_) + δ(CH_2_)	1672	ν(C = O) amide I
	4381	ν_a_(CH_2_) + δ(CH_2_)	1560	δ(C–N–H) amide II
	4604	1st overtone ν(CO) amide I + amide II	1455, 1405	δ(C–H)
	4893	ν_s_(NH) + δ(NH)		
	5157	ν(OH) + δ(OH)		
	5786	1st overtone ν(CH_2_)		
Gypsum [33]	4405	-	1622	δ(OH)
	4515	ν_s_(OH) + δ(OH	1178	ν_3_(SO_4_^2−^)
	5060sh	-	685, 602	ν_4_(SO_4_^2−^)
	5150	ν_a_(OH) + δ(OH)		
	5726	-		
Calcium carbonate [32]	-	-	1440	ν_3_(CO_3_^2−^)
	-	-	875	ν_2_(CO_3_^2−^)
	-	-	713	ν_4_(CO_3_^2−^)
Lead white [14,28]	4300	ν(OH) + deformation modes (Pb-OH)	1447	ν_3_(CO_3_^2−^)
	4403	ν(OH) + ν(CO)	1047	ν_1_(CO_3_^2−^)
	4988	-	680	ν_4_(CO_3_^2−^)
	5135	-		
Dammar [18,32]	4057	3rd overtone δ(CC)	1705	ν(C = O)
	4164	ν_s_(CH_2_) + δ(CH_2_)	1483, 1455	δ(CH)
	4345	ν_a_(CH_2_) + δ(CH_2_)		
	4632	1st overtone ν(CO) amide I + amide II		
	4830	ν(CO) + ν(OH)		
	5190	ν(OH) + δ(OH)		
	5737	1st overtone ν_a_(CH_2_)		
	5864	1st overtone ν_s_(CH_2_)		

## Data Availability

Not applicable.

## Supplementary Materials

The following supporting information can be downloaded at: https://www.mdpi.com/article/10.3390/s22052052/s1. Figure S1. FT-NIR spectrum of (a) calcite-based preparatory layer and b) animal glue. Figure S2. FT-NIR and MIR spectra of (a) *tempera grassa* with linseed oil, (b) *tempera grassa* with walnut oil, (c) tempera, (d) linseed oil on tempera, (e) walnut oil on tempera. The pigment is azurite. Legend: * = signals due to azurite. Figure S3. FT-NIR and MIR spectra of (a) *tempera grassa* with linseed oil, (b) *tempera grassa* with walnut oil, (c) tempera, (d) linseed oil on tempera, (e) walnut oil on tempera. The pigment is yellow ochre. Legend: * =signals due to yellow ochre. Figure S4. Comparison of NIR spectra of tempera and *tempera grassa* with yellow ochre (* = band of the pigment). The arrows indicate the signals of the lipid component, which increase in intensity in the mixed binders. Legend: (grey line) egg tempera; (dot line) *tempera grassa* with linseed oil; (black line) *tempera grassa* with walnut oil. Figure S5. Cross-section showing the thickness of the superficial layer of dammar varnish. Figure S6. FT-NIR and MIR spectra of: (a) white primer layer with walnut oil and lead white, (b) lead white in linseed oil on white primer layer, (c) azurite in linseed oil on white primer layer, (d) yellow ochre in linseed oil on white primer layer. Legend * = signals due to lead white; ∎ = signals due to azurite; • = signals due to yellow ochre. Figure S7. FTIR spectra of: (a) pink primer layer with lead white, red lead and thin yellow in walnut oil, (b) lead white in linseed oil on pink primer layer (c) azurite in linseed oil on pink primer layer, (d) yellow ochre in linseed oil on pink primer layer. Legend * = signals due to lead white; ∎ = signals due to azurite; • = signals due to yellow ochre. Figure S8. Loadings of the first principal component (a) and of the second principal component (d) of the NIR spectra (6000–4000 cm^−1^) of pure binders and of model painting samples on calcite ground layer. In the model samples, lead white (LW) was used as pigment. The first derivatives of the NIR spectra of lead white (b), linseed oil (c) and egg yolk (e) are shown for comparison. Figure S9. Score plot of the first two principal components of the NIR spectra (6000–4000 cm^−1^) of pure binders and of model painting samples on calcite ground layer. In the model samples, yellow ochre (O) was used as pigment. Figure S10. Score plot of the first two principal components of the NIR spectra (6000–4000 cm^−1^) of pure binders and of model painting samples on calcite ground layer. In the model samples, azurite (A) was used as pigment. A shift of the points corresponding to the spectra of the painting layers containing the blue pigment is due to the overlapping of its bands with those of oil. Figure S11. Loadings of the first principal component (a) and of the second principal component (c) of the NIR spectra (6000–4000 cm^−1^) of pure binders and of model painting samples on gypsum ground layer. In the model samples, lead white (LW) was used as pigment. The first derivatives of the NIR spectra of gypsum (b) and egg yolk (d) are shown for comparison. Figure S12. FT-NIR spectra of lead palmitate synthesised for comparison. Figure S13. Comparison between the FT-NIR spectrum of an oil layer as such on a glass slide (black line) and that of an oil layer on which a layer of synthetic varnish was applied (grey line). Figure S14. FT-MIR spectra from some details of the paintings: (a) “The Virgin and Child” by A. Boltraffio; (b) “The Virgin and Child” by F. Galli; (c) “The Virgin feeding the Child” Lombard school painter; (d) “St. Augustine and St. Jerome” by A. Bergognone; (e) “St. John the Baptist” by B. Zenale. In all cases, the signals are mainly due to synthetic restoration varnishes. Table S1. Assignments of NIR and MIR bands of azurite, yellow ochre, and hematite [45].

## References

[1] Matteini M., Moles A. (1989). La Chimica nel Restauro. I Materiali dell’arte Pittorica.

[2] Maltese C. (1993). Preparazione e Finitura delle opere pittoriche. Materiali e metodi. Preparazioni e imprimiture—Leganti -vernici—cornice.

[3] Ormsby B., Gottsegen M., Stoner J.H., Rushfield R. (2012). Grounds 1400-1900. Conservation of Easel Paintings.

[4] Vallance S.L. (1997). Critical Review: Applications of Chromatography in Art Conservation: Techniques Used for the Analysis and Identification of Proteinaceous and Gum Binding Media. Analyst.

[5] Castro R.M., Carbó M.T.D., Martínez V.P., Adelantado J.V.G., Reig F.B. (1997). Study of binding media in works of art by gas chromatographic analysis of amino acids and fatty acids derivatized with ethyl chloroformate. J. Chromatogr. A.

[6] Colombini M.P., Modugno F., Menicagli E., Fuoco R., Giacomelli A. (2000). GC-MS characterization of proteinaceous and lipid binders in UV aged polychrome artifacts. Microchem. J..

[7] Colombini M.P., Modugno F., Giacomelli M., Francesconi S. (1999). Characterisation of proteinaceous binders and drying oils in wall painting samples by gas chromatography–mass spectrometry. J. Chromatogr. A.

[8] Colombini M.P., Andreotti A., Bonaduce I., Modugno F., Ribechini E. (2010). Analytical Strategies for Characterizing Organic Paint Media Using Gas Chromatography/Mass Spectrometry. Acc. Chem. Res..

[9] Dey T., Carter J.C., Swift K. (2020). SEM-EDX and FTIR analysis of archaeological ceramic potteries from southern Italy. Microscopy.

[10] Prati S., Joseph E., Sciutto G., Mazzeo R. (2010). New Advances in the Application of FTIR Microscopy and Spectroscopy for the Characterization of Artistic Materials. Acc. Chem. Res..

[11] Chércoles Asensio R., San Andrés Moya M., De La Roja J.M., Gómez M. (2009). Analytical characterization of polymers used in conservation and restoration by ATR-FTIR spectroscopy. Anal. Bioanal. Chem..

[12] Glavcheva Z.I., Yancheva D.Y., Kancheva Y.K., Velcheva E.A., Stamboliyska B.A. (2014). Development of FTIR spectra database of reference art and archaeological materials. Bulg. Chem. Commun..

[13] Silva C.E., Silva L.P., Edwards H.G.M., De Oliveira L.F.C. (2006). Diffuse reflection FTIR spectral database of dyes and pigments. Anal. Bioanal. Chem..

[14] Zaffino C., Guglielmi V., Faraone S., Vinaccia A., Bruni S. (2015). Exploiting external reflection FTIR spectroscopy for the in-situ identification of pigments and binders in illuminated manuscripts. Brochantite and posnjakite as a case study. Spectrochim. Acta Part A Mol. Biomol. Spectrosc..

[15] Doménech Carbó M.T., Bosch Reig F., Gimeno Adelantado J.V., Periz Martínez V. (1996). Fourier transform infrared spectroscopy and the analytical study of works of art for purposes of diagnosis and conservation. Anal. Chim. Acta.

[16] Ozaki Y., Morita S., Morisawa Y., Ozaki Y., Huck C., Tsuchikawa S., Engelsen S.B. (2021). Spectral Analysis in the NIR Spectroscopy. Near-Infrared Spectroscopy Theory, Spectral Analysis, Instrumentation, and Applications.

[17] Olinger J.M., Griffiths P.R. (1988). Sample/spectrum relationships for resolution enhancement in near infrared reflectance spectrometry. Mikrochim. Acta.

[18] Vagnini M., Miliani C., Cartechini L., Rocchi P., Brunetti B.G., Sgamellotti A. (2009). FT-NIR spectroscopy for non-invasive identification of natural polymers and resins in easel paintings. Anal. Bioanal. Chem..

[19] Jurado-López A., Luque de Castro M.D. (2004). Use of near infrared spectroscopy in a study of binding media used in paintings. Anal. Bioanal. Chem..

[20] Carlesi S., Becucci M., Ricci M. (2017). Vibrational spectroscopies and chemometry for nondestructive identification and differentiation of painting binders. Hindawi J. Chem..

[21] Invernizzi C., Rovetta T., Licchelli M., Malagodi M. (2018). Mid and near-infrared reflection spectral database of natural organic materials in the cultural heritage field. Hindawi Int. J. Anal. Chem..

[22] Ricciardi P., Delaney J.K., Facini M., Zeibel J.G., Picollo M., Lomax S., Loew M. (2012). Near infrared reflectance imaging spectroscopy to map paint binders in situ on illuminated manuscripts. Angew. Chem. Int. Ed..

[23] Spring M., Mazzotta A., Roy A., Billinge R., Peggie D. (2011). Painting practice in Milan in the 1490s: The influence of Leonardo. Natl. Gallery Tech. Bull..

[24] Cennini C., Frezzato F. (2003). Il libro dell’arte.

[25] Spring M., Howard H., Kirby J., Padfield J., Peggie D., Roy A., Stephenson-Wright A. (2011). Studying Old Master Paintings, Technology and Practice, Proceedings of the National Gallery Technical Bulletin 30th Anniversary Conference Postprints.

[26] Dunkerton J., Spring M. (1998). The development of painting on coloured surfaces in sixteenth-century Italy. Stud. Conserv..

[27] Galli A., Gargano M., Bonizzoni L., Bruni S., Interlenghi M., Longoni M., Passaretti A., Caccia M., Salvatore C., Castiglioni I. (2021). Imaging and spectroscopic data combined to disclose the painting techniques and materials in the fifteenth century Leonardo atelier in Milan. Dyes Pigments.

[28] Miliani C., Rosi F., Daveri A., Brunetti B.G. (2012). Reflection infrared spectroscopy for the non-invasive in situ study of artists’ pigments. Appl. Phys. A.

[29] Vetter W., Latini I., Schreiner M. (2019). Azurite in medieval illuminated manuscripts: A reflection FTIR study concerning the characterization of binding media. Herit. Sci..

[30] Meilunas R.J., Bentsen J.G., Steinberg A. (1990). Analysis of aged paint binders by FTIR spectroscopy. Stud. Conserv..

[31] Ciofini D., Striova J., Camaiti M., Siano S. (2016). Photo-oxidative kinetics of solvent and oil-based terpenoid varnishes. Polym. Degrad. Stabil..

[32] Derrick M.R., Stulik D.C., Landry J.M. (1999). Infrared Spectroscopy in Conservation Science.

[33] Rosi F., Daveri A., Doherty B., Nazzareni S., Brunetti B.G., Sgamellotti A., Miliani C. (2010). On the use of overtone and combination bands for the analysis of the CaSO4–H2O system by mid-infrared reflection spectroscopy. Appl. Spectrosc..

[34] Bacci M., Baronti S., Casini A., Castagna P., Linari R., Orlando A., Picollo M., Radicati B. (1995). Detection of alteration products in artworks by non-destructive spectroscopic analysis. Mat. Res. Soc. Symp. Proc..

[35] Mazzeo R., Prati S., Quaranta M., Joseph E., Kendix E., Galeotti M. (2008). Attenuated total reflection micro FTIR characterisation of pigment–binder interaction in reconstructed paint films. Anal. Bioanal. Chem..

[36] Museo Poldi Pezzoli—Artworks. https://museopoldipezzoli.it/catalogo/#/dettaglio/119305_Madonna%20con%20Bambino.

[37] Beccaria C., Menu M. (2014). Some observations on the painting technique of Boltraffio’s The Madonna and Child. Leonardo da Vinci’s Technical Practice: Paintings, Drawing and Influence: Paintings, Drawings and Influence.

[38] Pinacoteca di Brera—The Collection Online. https://pinacotecabrera.org/collezione-online/opere/madonna-col-bambino-napoletano/.

[39] Museo Poldi Pezzoli—Artworks. https://museopoldipezzoli.it/catalogo/#/dettaglio/121656_Madonna%20che%20allatta%20il%20Bambino.

[40] Gallone A. (1998). Scuola leonardesca “Madonna col Bambino” (Museo Poldi Pezzoli). Analisi di Alcuni Campioni di Colore.

[41] Ministero dei Beni Culturali (Italia)—Catalogo generale dei Beni Culturali. https://catalogo.beniculturali.it/detail/HistoricOrArtisticProperty/0300702290-3.

[42] Old Masters including Portrait Miniatures from the Pohl-Ströher Collection. https://www.sothebys.com/en/buy/auction/2020/old-masters-including-portrait-miniatures-from-the-pohl-stroeher-collection/bernardo-zenale-saint-john-the-baptist-standing-in.

[43] Accademia Carrara—The Collection. https://www.lacarrara.it/catalogo/81lc00236/?highlight=andrea%20solario%20ecce%20homo.

[44] Li X., Sun C., Zhou B., He Y. (2015). Determination of Hemicellulose, Cellulose and Lignin in Moso Bamboo by Near Infrared Spectroscopy. Sci Rep..

[45] Farmer V.C. (1974). The Infrared Spectra of Minerals.

