# Supplementary material for: FT-NIR Spectroscopy for the Non-Invasive Study of Binders and Multi-Layered Structures in Ancient Paintings: Artworks of the Lombard Renaissance as Case Studies
Source: Sensors (Basel). 2022 Mar 6;22(5):2052. doi: 10.3390/s22052052 (PMC8914679; doi:10.3390/s22052052)
Supplement: Supplementary file 1 [file sensors-22-02052-s001.zip › sensors-1556761-supplementary.pdf]

Supplementary Materials

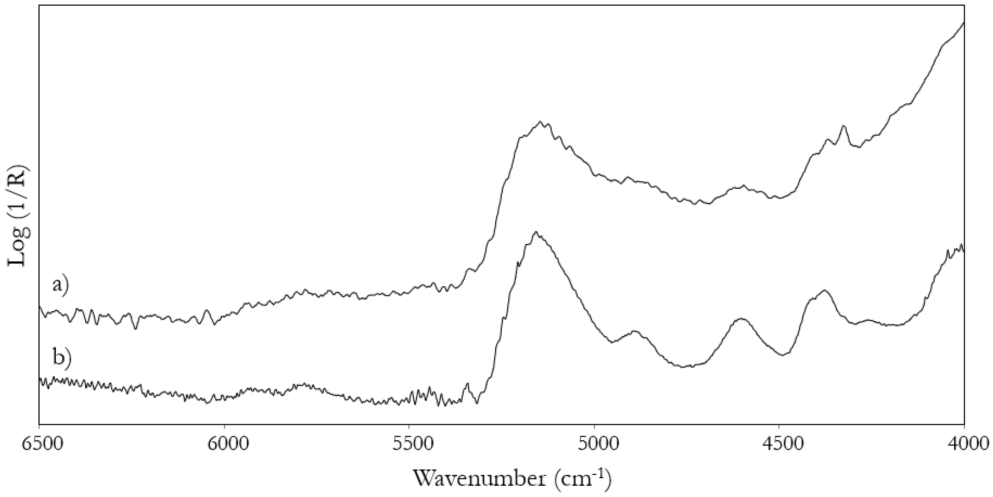

Figure S1. FT-NIR spectrum of (**a**) calcite-based preparatory layer and (**b**) animal glue.

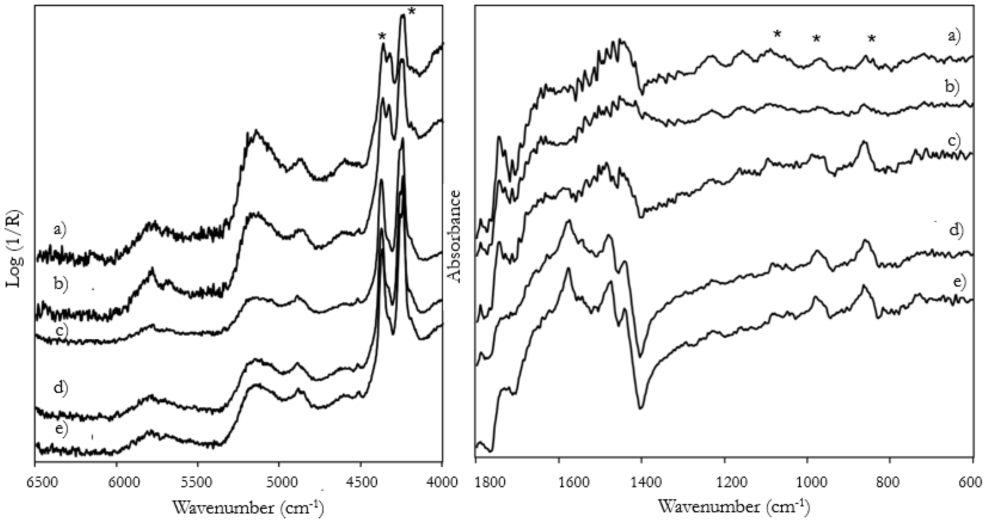

Figure S2. FT-NIR and MIR spectra of (**a**) *tempera grassa* with linseed oil, (**b**) *tempera grassa* with walnut oil, (**c**) tempera, (**d**) linseed oil on tempera, (**e**) walnut oil on tempera. The pigment is azurite. Legend: * = signals due to azurite.

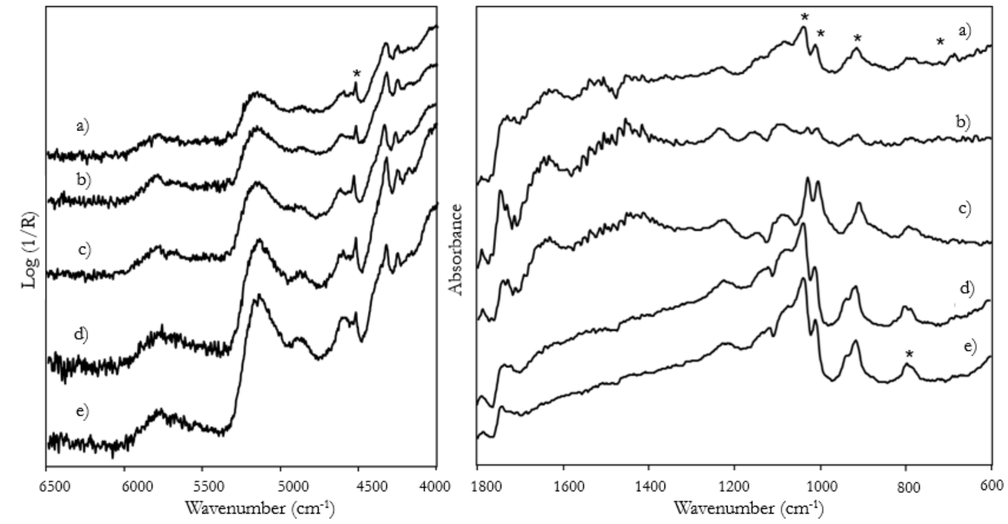

Figure S3. FT-NIR and MIR spectra of (**a**) *tempera grassa* with linseed oil, (**b**) *tempera grassa* with walnut oil, (**c**) tempera, (**d**) linseed oil on tempera, (**e**) walnut oil on tempera. The pigment is yellow ochre. Legend: * =signals due to yellow ochre.

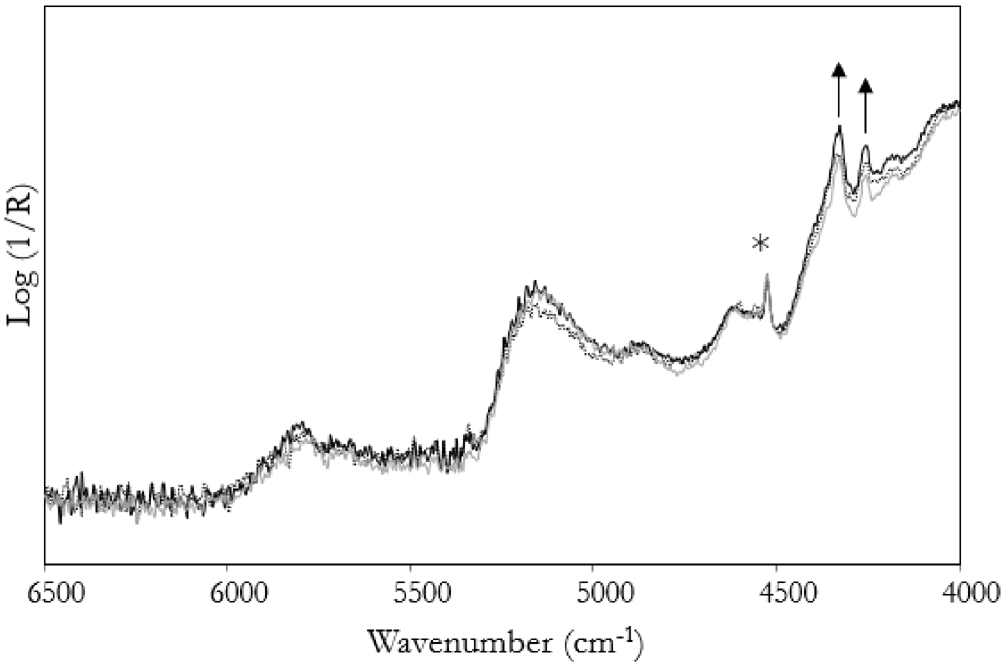

Figure S4. Comparison of NIR spectra of tempera and *tempera grassa* with yellow ochre (* = band of the pigment). The arrows indicate the signals of the lipid component, which increase in intensity in the mixed binders. Legend: (gray line) egg tempera; (dot line) *tempera grassa* with linseed oil; (black line) *tempera grassa* with walnut oil.

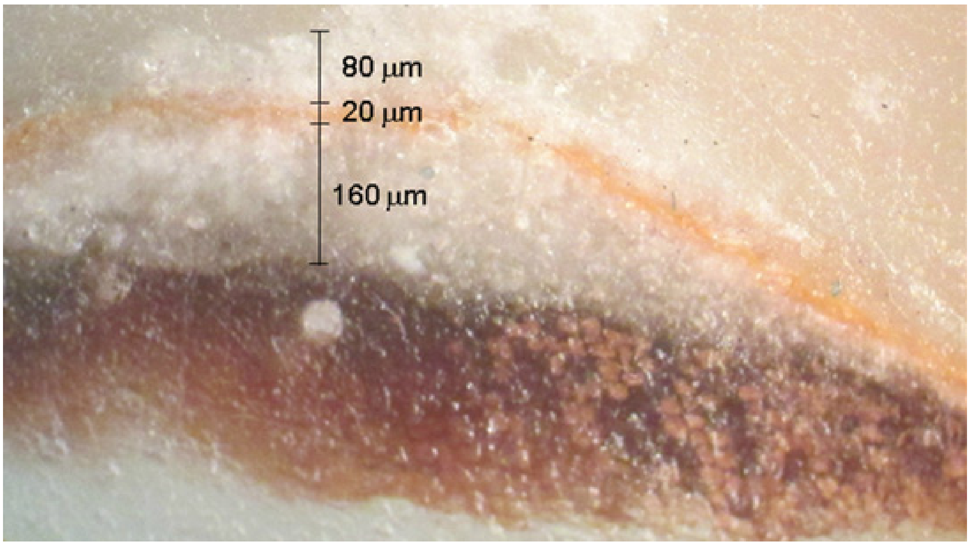

Figure S5. Cross section showing the thickness of the superficial layer of dammar varnish.

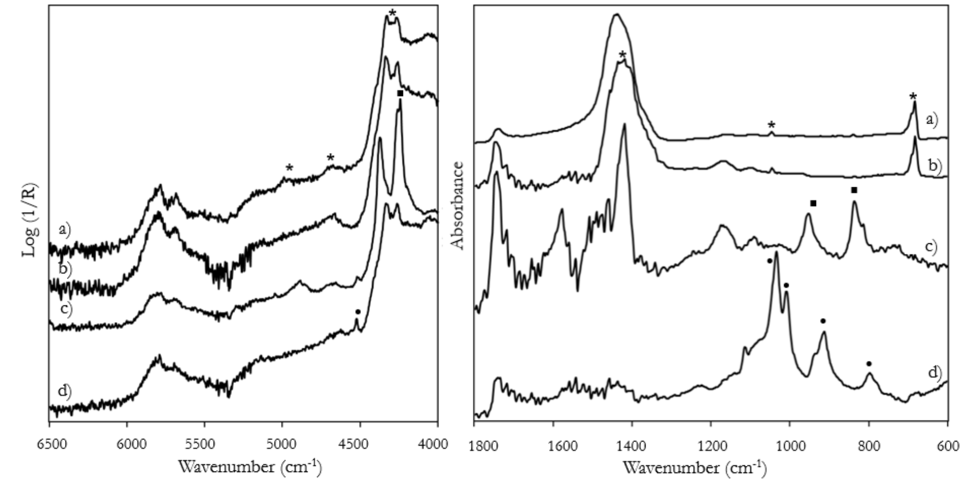

Figure S6. FT-NIR and MIR spectra of: (**a**) white primer layer with walnut oil and lead white, (**b**) lead white in linseed oil on white primer layer, (**c**) azurite in linseed oil on white primer layer, (**d**) yellow ochre in linseed oil on white primer layer. Legend * = signals due to lead white; ■ = signals due to azurite; • = signals due to yellow ochre.

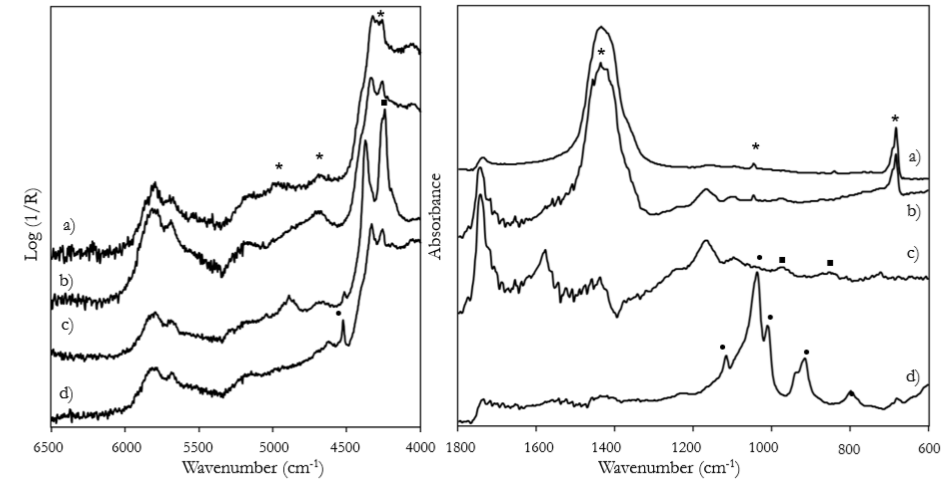

Figure S7. FTIR spectra of: (**a**) pink primer layer with lead white, red lead and thin yellow in walnut oil, (**b**) lead white in linseed oil on pink primer layer (**c**) azurite in linseed oil on pink primer layer,

(**d**) yellow ochre in linseed oil on pink primer layer. Legend * = signals due to lead white; ■ = signals due to azurite; • = signals due to yellow ochre.

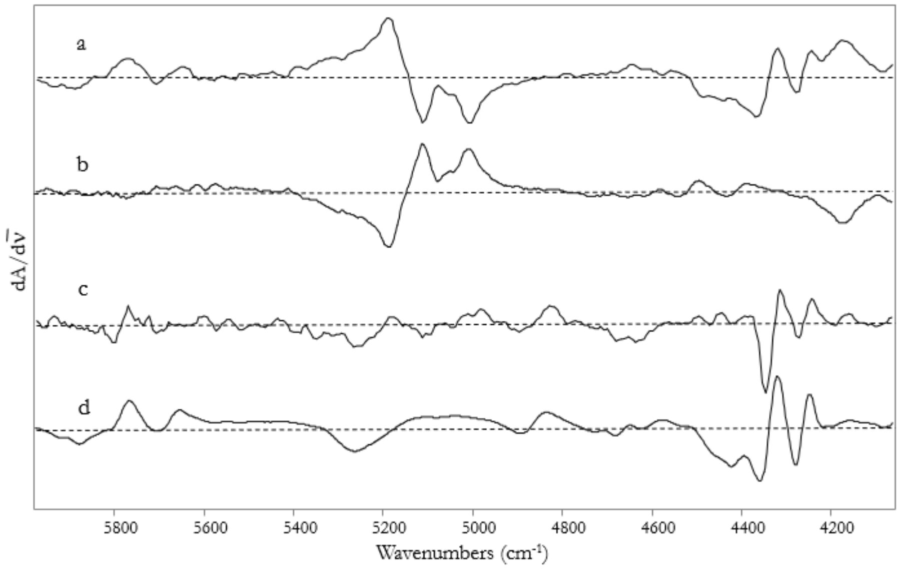

Figure S8. Loadings of the first principal component (**a**) and of the second principal component (**d**) of the NIR spectra (6000–4000 cm^{-1}) of pure binders and of model painting samples on calcite ground layer. In the model samples lead white (LW) was used as pigment. The first derivatives of the NIR spectra of lead white (**b**), linseed oil (**c**) and egg yolk (**e**) are shown for comparison.

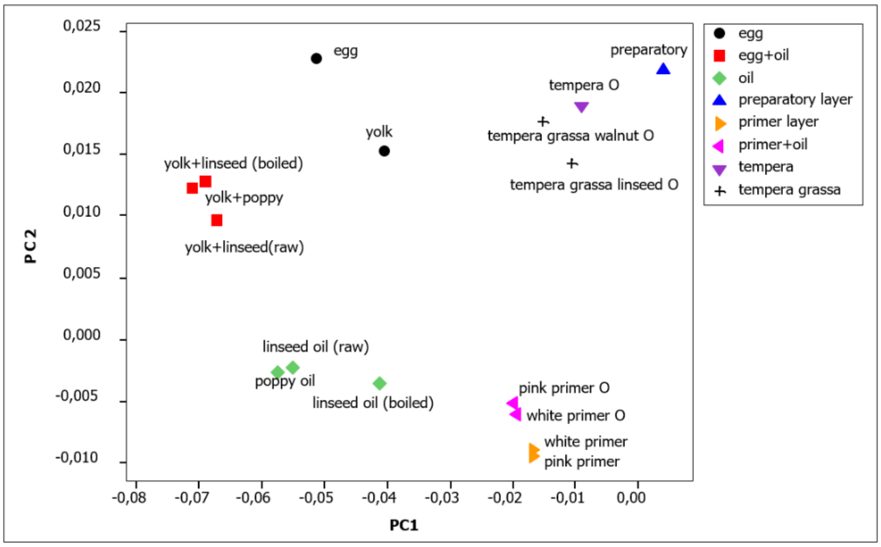

Figure S9. Score plot of the first two principal components of the NIR spectra (6000–4000 cm^{-1}) of pure binders and of model painting samples on calcite ground layer. In the model samples yellow ochre (O) was used as pigment.

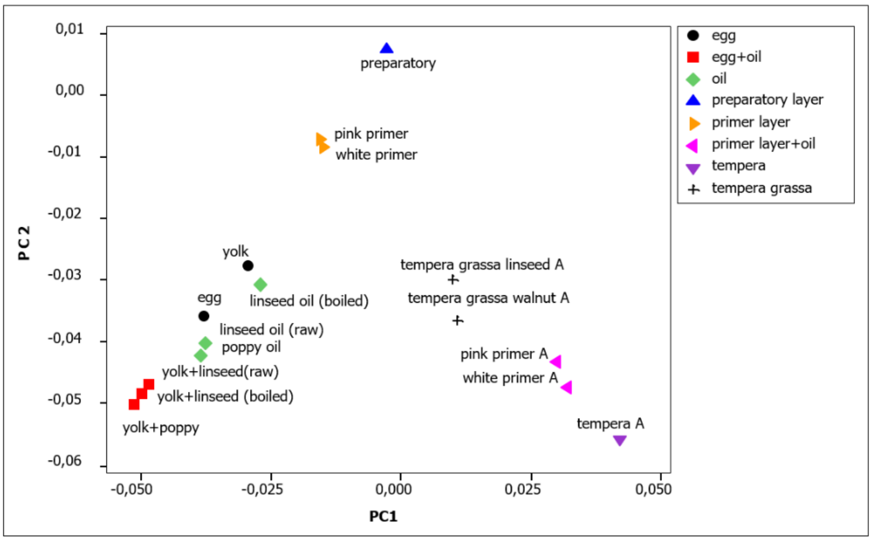

Figure S10. Score plot of the first two principal components of the NIR spectra (6000–4000 cm^{-1}) of pure binders and of model painting samples on calcite ground layer. In the model samples azurite (A) was used as pigment. A shift of the points corresponding to the spectra of the painting layers containing the blue pigment is due to the overlapping of its bands with those of oil.

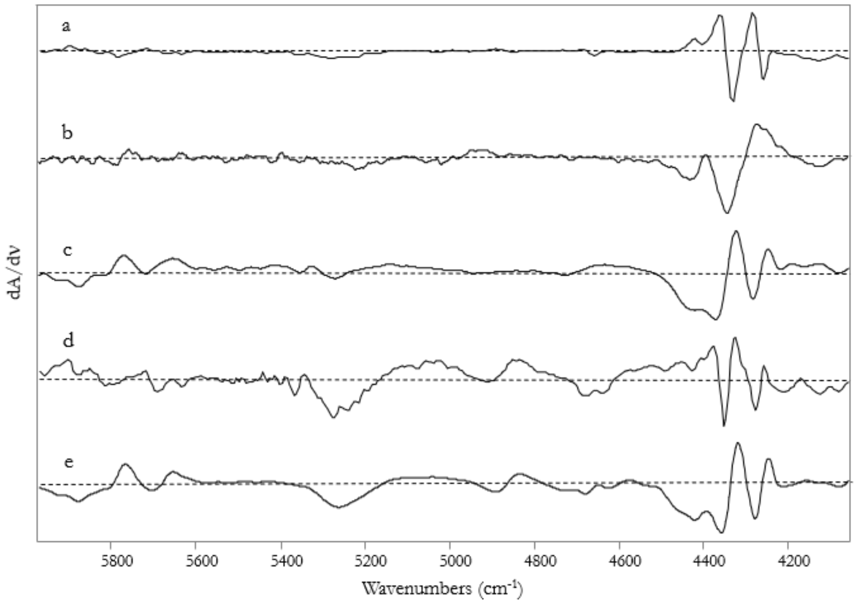

Figure S11. Loadings of the first principal component (**a**) and of the second principal component (**c**) of the NIR spectra (6000–4000 cm^{-1}) of pure binders and of model painting samples on gypsum ground layer. In the model samples lead white (LW) was used as pigment. The first derivatives of the NIR spectra of gypsum (**b**) and egg yolk (**d**) are shown for comparison.

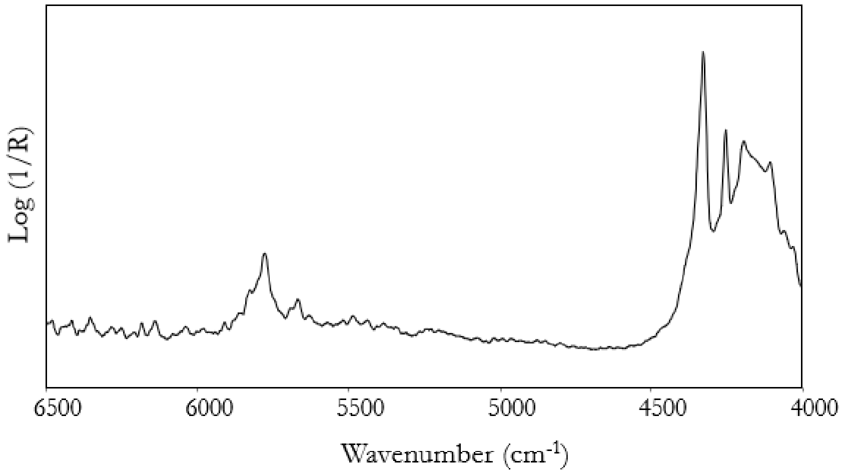

Figure S12. FT-NIR spectra of lead palmitate synthesised for comparison.

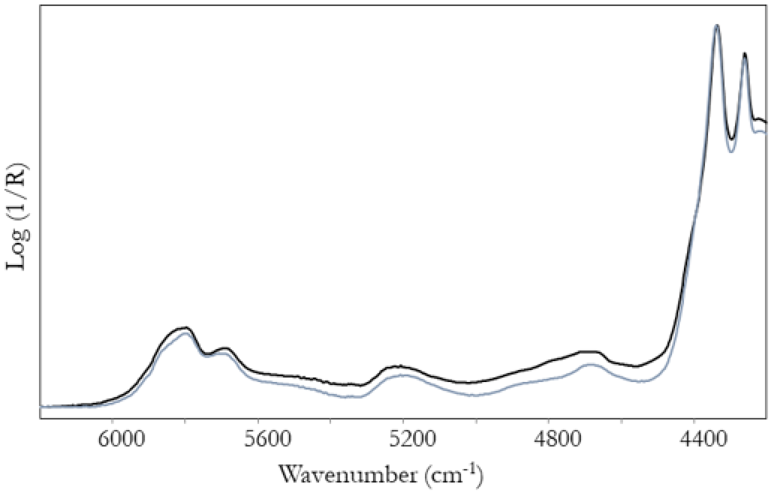

Figure S13. Comparison between the FT-NIR spectrum of an oil layer as such on a glass slide (black line) and that of an oil layer on which a layer of synthetic varnish was applied (grey line).

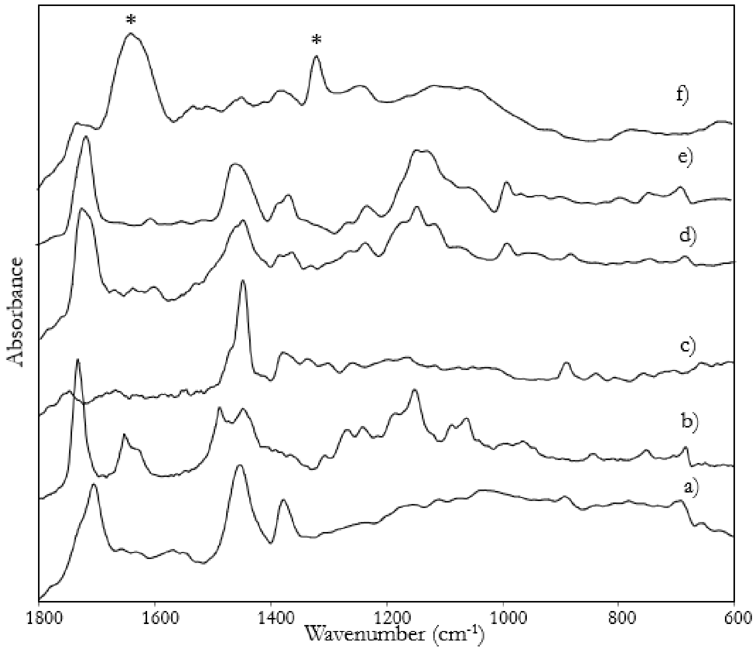

Figure S14. FT-MIR spectra from some details of the paintings: (**a**) "The Virgin and Child" by A. Boltraffio; (**b**) "The Virgin and Child" by F. Galli; (**c**) "The Virgin feeding the Child" Lombard school painter; (**d**) "St. Augustine and St. Jerome" by A. Bergognone; (**e**) "St. John the Baptist" by B. Zenale and (**f**) "Ecce Homo" by A. Solario. In all spectra, with the exception of spectrum (**f**), the signals are mainly due to synthetic restoration varnishes. * = bands due to calcium oxalate.

Table S1. Assignments of NIR and MIR bands of azurite, yellow ochre and hematite.

**Azurite** [1]	4235	2nd overtone $\nu_3(CO_3^{2-})$	1460, 1431	$\nu_3(CO_3^{2-})$
	4370	$\nu(OH) + \delta(OH)$	1090	$\nu_1(CO_3^{2-})$
	4520	-	960	$\delta(OH)$
	4895	-	840	$\nu_2(CO_3^{2-})$
	5047	-	500	ν(Cu–O)
	5298	-	460	ν(Cu–OH)
**Yellow ochre** [1, 2]	4525	$\nu(OH) + \delta(OH)$	1116, 1046	ν(Si–O–Si) kaolin
			915	δ(OH) kaolin
			801	γ(OH) goethite
			536	δ(Si–O–Al)
			473	δ(Si–O)
**Hematite** [2]	-	-	600, 483	Lattice vibrations

References

1. Zaffino, C.; Guglielmi, V.; Faraone, S.; Vinaccia, A.; Bruni, S. Exploiting external reflection FTIR spectroscopy for the in-situ identification of pigments and binders in illuminated manuscripts. Brochantite and posnjakite as a case study. *Spectrochim. Acta Part A Mol. Biomol. Spectrosc.* **2015**, *136*, 1076–1085. https://doi.org/10.1016/j.saa.2014.09.132.
2. Farmer, V.C. *The Infrared Spectra of Minerals*; Mineralogical Society, London, UK, 1974.
